# Lack of the Matricellular Protein SPARC (Secreted Protein, Acidic and Rich in Cysteine) Attenuates Liver Fibrogenesis in Mice

**DOI:** 10.1371/journal.pone.0054962

**Published:** 2013-02-11

**Authors:** Catalina Atorrasagasti, Estanislao Peixoto, Jorge B. Aquino, Néstor Kippes, Mariana Malvicini, Laura Alaniz, Mariana Garcia, Flavia Piccioni, Esteban J. Fiore, Juan Bayo, Ramón Bataller, Elizabeth Guruceaga, Fernando Corrales, Osvaldo Podhajcer, Guillermo Mazzolini

**Affiliations:** 1 Gene Therapy Laboratory, School of Medicine, Austral University. Derqui-Pilar, Buenos Aires, Argentina; 2 CONICET (Consejo Nacional de Investigaciones Científicas y Técnicas), Buenos Aires, Argentina; 3 Department of Medicine and Nutrition, University of North Carolina, Chapel Hill, North Carolina, United States of America; 4 Centro de Investigación Médica Aplicada, Universidad de Navarra, Pamplona, España; 5 Gene Therapy Laboratory, Fundación Instituto Leloir, Buenos Aires, Argentina; Institute of Hepatology, Foundation for Liver Research, United Kingdom

## Abstract

**Introduction:**

Secreted Protein, Acidic and Rich in Cysteine (SPARC) is a matricellular protein involved in many biological processes and found over-expressed in cirrhotic livers. By mean of a genetic approach we herein provide evidence from different *in vivo* liver disease models suggesting a profibrogenic role for SPARC.

**Methods:**

Two *in vivo* models of liver fibrosis, based on TAA administration and bile duct ligation, were developed on SPARC wild-type (SPARC^+/+^) and knock-out (SPARC^−/−^) mice. Hepatic SPARC expression was analyzed by qPCR. Fibrosis was assessed by Sirius Red staining, and the maturation state of collagen fibers was analyzed using polarized light. Necroinflammatory activity was evaluated by applying the Knodell score and liver inflammatory infiltration was characterized by immunohistochemistry. Hepatic stellate cell activation was assessed by α-SMA immunohistochemistry. In addition, pro-fibrogenic genes and inflammatory cytokines were measured by qPCR and/or ELISA. Liver gene expression profile was analyzed in SPARC^−/−^ and SPARC^+/+^ mice using Affymetrix Mouse Gene ST 1.0 array.

**Results:**

SPARC expression was found induced in fibrotic livers of mouse and human. SPARC^−/−^ mice showed a reduction in the degree of inflammation, mainly CD4+ cells, and fibrosis. Consistently, collagen deposits and mRNA expression levels were decreased in SPARC^−/−^ mice when compared to SPARC^+/+^ mice; in addition, MMP-2 expression was increased in SPARC^−/−^ mice. A reduction in the number of activated myofibroblasts was observed. Moreover, TGF-β1 expression levels were down-regulated in the liver as well as in the serum of TAA-treated knock-out animals. Ingenuity Pathway Analysis (IPA) analysis suggested several gene networks which might involve protective mechanisms of SPARC deficiency against liver fibrogenesis and a better established machinery to repair DNA and detoxify from external chemical stimuli.

**Conclusions:**

Overall our data suggest that SPARC plays a significant role in liver fibrogenesis. Interventions to inhibit SPARC expression are suggested as promising approaches for liver fibrosis treatment.

## Introduction

Secreted protein, acidic and rich in cysteine (SPARC), also called osteonectin or BM-40, is a secreted multifunctional extracellular matrix (ECM)-associated protein involved in a number of biological processes [1], [2]. Among other functions, SPARC plays a major role in the wound healing response to injury and tissue remodeling [1]. Regarding mechanisms likely therein involved, locally produced SPARC was found to stimulate collagen deposition, inflammatory cells recruitment, TGF-β1 production, mesenchymal cell proliferation and ECM proteins synthesis, in the context of kidney, skin and/or lung fibrogenesis [3], [4], while no studies were performed on liver fibrosis models. Due to its biological properties, SPARC was proposed as a therapeutic target to prevent fibrosis in chronic inflammatory and profibrogenic conditions [5].

Although SPARC is constitutively expressed in the liver under non-pathological conditions [6], it was found upregulated in fibrotic-related liver diseases such as cirrhosis [7], [8] and hepatocellular carcinoma [9], [10], [11]. During liver fibrogenesis, SPARC was found overexpressed in activated hepatic stellate (HSCs) and in endothelial cells [6], [7]. These findings suggest that SPARC may have a prominent role in liver fibrogenesis; moreover, we have recently demonstrated that a forced transitory reduction in SPARC expression levels by an adenovirus encoding an antisense specific for SPARC mRNA (AdasSPARC) attenuates fibrosis development in an *in vivo* experimental rat model [5].

During liver fibrogenesis TGF-β1 expression is induced. This cytokine plays a key role in the activation of HSCs and in the development of hepatic fibrosis [12]. Thus, different molecular strategies have been explored to block/reduce TGF-β1 mediated mechanisms including gene transfer of truncated TGF-β1 receptor type II or administration of a soluble TGF-β1 type II receptor, [13], [14]. Interestingly, a positive feedback between SPARC and TGF-β1 has been previously reported [3], [15].

To further elucidate the role of SPARC in hepatic fibrogenesis, we have herein used different *in vivo* disease models, i.e. involving either hepatotoxicity or biliary duct obstruction, in SPARC genetically deficient mice. Liver fibrosis development was found markedly attenuated in SPARC^−/−^ when compared to SPARC^+/+^ mice. Our data suggest that SPARC plays a major role in the pathogenesis of liver fibrosis, through myofibroblast recruitment/activation and induction of TGF-β1 expression. Additionally, microarray analyses likely involve DNA protective and repair mechanisms. Overall these results give further support to new therapeutic approaches based on SPARC expression inhibition for the treatment of patients with chronic liver diseases.

## Materials and Methods

### Animals and Experimental Design

Male C57BL/6x129SvJ (The Jackson Laboratory, Bar Harbor, Maine, USA) SPARC^+/+^ and SPARC^−/−^ mice (2–3 months-old) were used. In a hepatotoxic model, animals were administered intraperitoneally (i.p.) with 200 mg/kg of thioacetamide (TAA) (Sigma, St Louis, MO), 3 times a week as described previously [16], [17]. Animals were sacrificed at 2 and 10 weeks after TAA application onset and blood and liver samples were obtained. In a cholestasis model, mice were subjected to bile duct ligation (BDL) or sham-operation or they were left untreated. For surgeries, animals were anesthetized with sodium pentobarbital. A midline laparotomy was performed and the common bile duct was doubly ligated with 4–0 silk. Sham operation procedure was similar but without ligating the bile duct. Animals were sacrificed at 7 days after surgery and blood and liver samples were obtained. All experimental procedures were performed according to the “Guide for the Care and Use of Laboratory Animals” published by the U.S. National Research Council (National Academy Press, Washington, D.C. 1996) and approved by the School of Medicine, Austral University (Permission number: FBA002).

### Human Liver Specimens

A total of 7 liver biopsies, from 2 non-cirrhotic and 5 cirrhotic subjects were used in real-time polymerase chain reaction studies. Cirrhosis etiology was diverse (primary biliary cirrhosis, biliary atresia, hepatitis C virus (HCV) infection, hemochromatosis and cryptogenic; patients #1 to #5). All participants gave their written informed consent to the study, which was approved by the Institutional Ethics Committee and by the Ministry of Health of Buenos Aires State (Permission number: 2919/179/2011).

### Reverse Transcription-polymerase Chain Reaction (RT-PCR)

Liver tissue was homogenized and total RNA was extracted by using Trizol Reagent (Sigma-Aldrich Co., St. Louis, MO). Total RNA (2 µg) was reverse transcribed with 200 U of SuperScript II Reverse Transcriptase (Invitrogen, Carlsbad, CA) using 500 ng of Oligo (dT) primers. cDNAs were subjected to real-time polymerase chain reaction (qPCR) (Stratagene Mx3005p, Stratagene, La Jolla, CA, USA). For qPCR of mouse samples the mRNA levels of SPARC, alpha 2-type I collagen (COL1A2), matrix metalloproteinase-2 (MMP-2) and transforming growth factor-β1 (TGF-β1) were quantified by SYBR® Green (Invitrogen), using the following primers: SPARC sense (5′-CCACACGTTTCTTTGAGACC-3′); SPARC antisense (5′-GATGTCCTGCTCCTTGATGC-3′), COL1A2, 5′-CCTACATGGACCAGCAGACTG -3 (forward), 5′- GGAGGTCTTGGTGGTTTTGTA -3′ (reverse), TGF-β1 5′-CCACTCGCTTCTTTGAGACC-3′ (forward), 5′-TAGTGGAAGTGGGTGGGGAC-3′ (reverse) and MMP-2 5′-CTCAGATCCGTGGTGAGAT -3 (forward), 5′-AGGCTGGTCAGTGGCTTGG-3′ (reverse). For qPCR of human samples, the following primers were used: SPARC, 5′-AAACCGAAGAGGAGGTGGTG-3′ (forward), 5′-GCAAAGAAGTGGCAGGAAGA-3′ (reverse). All PCR amplifications were carried out using a cycle of 95°C for 10 min and 40 cycles under the following parameters: 95°C for 30 sec, corresponding melting temperature for 30 sec, 72°C for 1 min. At the end of the PCR reaction, the temperature was increased from 60°C to 95°C at a rate of 2°C/min, and the fluorescence was measured every 15 sec to construct the melting curve. Values were normalized to levels of glyceraldehyde-3-phosphate dehydrogenase (GAPDH; used as housekeeping) transcript (forward 5′-CATCTCTGCCCCCTCTGCTG -3′; reverse 5′-GCCTGCTTCACCACCTTCTTG-3′). Data were processed by the ΔΔCt method. The relative amount of the PCR product amplified from untreated cells was set as 1. A non-template control (NTC) was run in every assay, and all determinations were performed in triplicate in two or three separated experiments.

### Pathology, Immunofluorescence and Immunohistochemistry Studies

Some harvested livers were immersed in 10% phosphate-buffered formalin. Fixed tissue was embedded in paraffin, sectioned (5 µm) and hematoxylin-eosin (H&E), Masson-trichrome or Sirius red stained, or used for immunohistochemical analysis of α-SMA expression. The Knodell score was used to grade the severity of the necroinflammatory process and fibrosis [18]. Assessments were blinded performed by an experienced pathologist. For CD4 immunohistochemistry, tissue was embedded in OCT, cryostat sectioned and fixed for 15 min in alcoholic formalin. In brief, after tissue dehydration endogenous peroxidase was blocked with 3% H_2_O_2_ in 95% ethanol. Thereafter, sections were subsequently blocked for endogenous biotin and avidin (Blocking kit, Vector Laboratories Inc.) and sections were incubated with the primary antibody anti-CD4 (1∶75, 0.2% BSA in PBS) overnight. After being washed in phosphate-buffered saline, slides were incubated with peroxidase-linked biotinylated goat anti-mouse secondary antibody (1∶100, Vector Laboratories Inc.) for 2 h, washed and further incubated with AB complex at RT. They were then washed twice with PBS and twice with 0.1 M acetate buffer before incubation with a solution of 3.3-diaminobenzidine (DAB; Sigma), ammonium nickel sulfate and H_2_O_2_ until signal was developed.

For immunofluorescence SPARC analysis, mice were perfused with 4% paraformaldehyde and liver tissue was dissected out, post-fixed for 90 minutes and subsequently placed in 10 and 20% sucrose. For SPARC immunofluorescence assay, samples were embedded in OCT and cryostat sectioned (12 µm). After a 1 hour incubation in blockage buffer (5% normal donkey serum -Jackson ImmunoResearch, PA, USA), 1% BSA, 0.3% Triton-X in 1×PBS; room temperature), tissue was incubated with a rat anti-SPARC monoclonal antibody (1∶150, 0.1% BSA, 0.3% Triton-X, 0.02% sodium azide in PBS; overnight, 4°C; R&D, MN, USA), together with either rabbit anti-mouse α-SMA (1∶75; ab5694, Abcam, MA, USA) or rabbit anti-Von Willebrand factor (vWF; 1∶215; Sigma, MO, USA) polyclonal antibodies. After extensive washing, tissue was incubated with Cy3-conjugated donkey anti-rat IgG and FITC-conjugated donkey anti-rabbit IgG secondary antibodies (1∶450; 1% BSA in PBS, 2 hours, room temperature; Jackson ImmunoResearch). Images were captured using a Nikon C1 laser confocal microscope.

For chromogenic immunohistochemical analysis and quantification of α-SMA expression, sections were deparaffinized, rehydrated and heated with buffer citrate (pH = 6) in a microwave protocol. Endogenous peroxidase was blocked with 3% H_2_O_2_ in 95% ethanol. Thereafter, sections were subsequently blocked for endogenous biotin and avidin (Blocking kit, Vector Laboratories Inc.), and for unspecific binding of the primary antibody (1% BSA-PBS). Tissue was then incubated with the rabbit anti-mouse α-SMA polyclonal antibody (1∶100; Abcam). After extensive washing, slides were incubated with peroxidase-linked biotinylated goat anti-rabbit secondary antibodies for 2 h, washed and further incubated with AB complex at RT. They were then washed twice with PBS and twice with 0.1 M acetate buffer before incubation with a solution of 3.3-diaminobenzidine (DAB; Sigma), ammonium nickel sulfate and H_2_O_2_ until signal was developed. Primary antibody incubation was omitted in control slides, only rendering a faint staining (not shown). Quantitative analysis of α-SMA immunostained area was performed by computerized morphometric analysis (CMA). About 80 light microscope images (200X) per specimen were captured and analyzed using a color threshold detection system developed in ImageJ software (NIH, USA). Results were expressed as percentage of positive area.

### Quantification of Hepatic Collagen Content, Collagen Fibers Maturation and Hyaluronan Deposition

Quantitative analysis of collagen content was performed by CMA on samples stained with Sirius red. For this purpose, randomly sampled two hundred light microscope images (200X) per liver specimen, excepting large centrilobular veins and large portal tracts (≥150 µm) were analyzed. About 80 light microscope images (200X) per specimen were captured and analyzed using a color threshold detection system developed in ImageJ (National Institutes of Health). Values are expressed as percentage of positive area. To assess the degree of packaging of collagen fibers and their maturation state, liver sections were examined with polarized light microscopy using an Olympus BX60 microscope (Olympus, Tokyo, Japan). Hyaluronan staining was performed as previously described [19], [20].

### ELISA Assay of TGF-β1

Total TGF-β1 serum levels after 10 weeks of TAA treatment were measured by ELISA (R&D systems, Minneapolis, MN, USA), following manufacturer recommendations. To convert all latent TGF-β1 to the active form, samples were pretreated with 1 M HCl for 15 min at room temperature. Finally, the reaction was stopped, by 1 M NaOH neutralization, and the optical density of each well was determined at 540 or 570 nm.

### Microarray Data Analysis

Samples were processed following Affymetrix recommendations and cRNA was hybridized to the Affymetrix Mouse Gene ST 1.0 array. Both background correction and normalization were done using RMA (Robust Multichip Average) algorithm [21]. Then, a filtering process was performed to eliminate low expression probe sets. Applying the criterion of an expression value greater than 64 in 2 samples for each experimental condition, 18187 probesets were selected for statistical analysis. R and Bioconductor were used for preprocessing and statistical analysis. LIMMA (Linear Models for Microarray Data) [22], [23] was used to find out the probe sets that showed significant differential expression between experimental conditions. Genes were selected as significant using a criteria of p-value <0.01.

### Functional and Pathway Analysis

Functional enrichment analysis of Gene Ontology (GO) categories was carried out using standard hypergeometric test [24]. The biological knowledge extraction was complemented through the use of Ingenuity Pathway Analysis (Ingenuity Systems, www.ingenuity.com), which database includes manually curated and fully traceable data derived from literature sources.

### Transaminases Measurement

Serum alanine (ALT) and aspartate (ALT) transaminases were measured using an ARCHITECT® (Abbott) autoanalyzer.

### Migration Assay

SPARC^+/+^ and SPARC^−/−^ mice were sacrificed, spleen were excised, and single cell suspensions were prepared. Cell suspension were treated with RBC lysis buffer (0.15 mol/L NH_4_Cl, 1 mmol/L KHCO_3_, 0.1 mmol/L Na_2_EDTA) and washed with PBS 1% bovine serum albumin. Then, cells were plated in a plastic petri dish for 15 min and finally counted and subjected to migration assay. Migratory capacity to rCCL19 (Peprotech) was assayed using a 48-Transwell microchemotaxis Boyden Chamber unit (Neuroprobe, Inc.). In brief, splenocytes (20,000 cells/well) were placed in the upper chamber of the Transwell unit, which was separated from the lower chamber by 8 µm pore polycarbonate filters (Nucleopore membrane, Neuroprobe). rCCL19 (10 ng/µl) was placed in the lower chamber of the Transwell unit. All the system units were incubated for 2 h at 37°C in a 5% CO_2_ humidified atmosphere. After that, the membrane was carefully removed and cells on the upper side of the membrane were scraped off with a blade. Cells attached to the lower side of the membrane were fixed in 2% formaldehyde, and the membranes from the microchemotaxis Boyden Chamber unit were stained with 40.6 diamidino-2-phenylindole dihydrochloride (DAPI, Sigma-Aldrich). Cells were counted using fluorescent-field microscopy and a 10x objective lens: the images captured in three representative visual fields were analyzed using CellProfiler software (www.cellprofiler.com), and the mean number of cells/field (SEM was calculated).

### Statistical Analysis

Data are expressed as mean ± SEM when appropriate. Statistical analysis was performed using Student’s *t* test or Mann-Whitney, according to value data distribution. Differences were considered to be significant when p<0.05.

## Results

### Expression of SPARC during Liver Fibrogenesis

**Figure 1:**
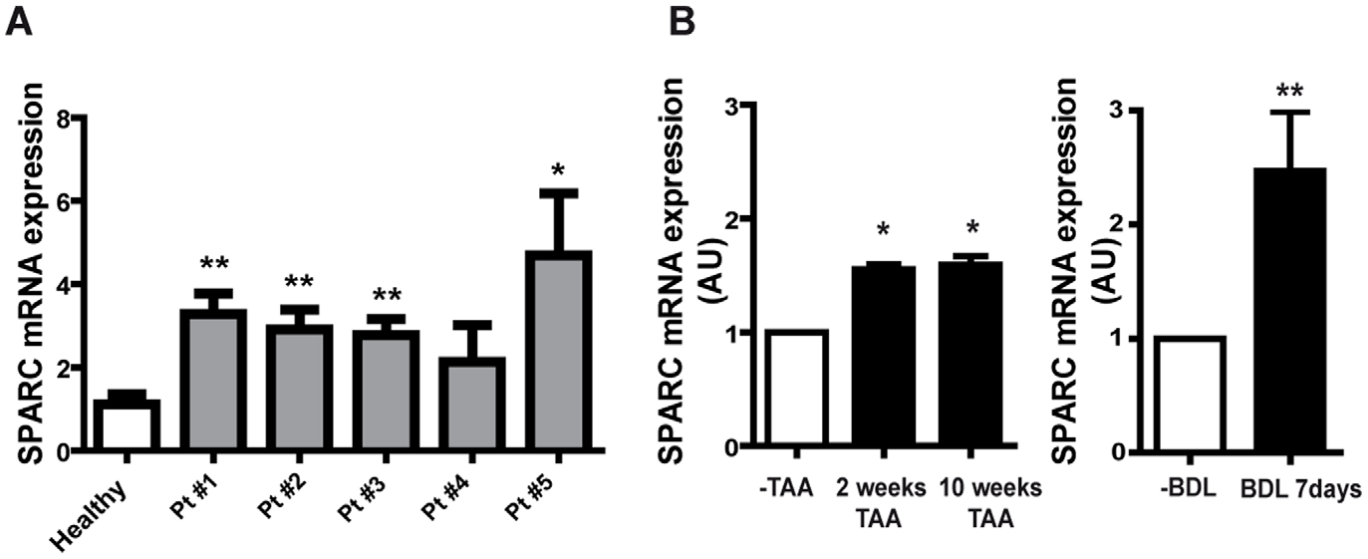
Induction of SPARC mRNA expression during liver fibrogenesis. (A) Quantitative data showing differences in SPARC mRNA expression levels in human cirrhotic (Pt#1 to Pt#5) with fibrosis degree F4 and non-cirrhotic liver samples as measured by qPCR. *p<0.05, **p<0.01 compared with healthy liver samples. Mann-Whitney test. (B) qPCR analyses of liver samples from TAA and BDL mice. Complementary DNA was synthesized and was subjected to qPCR for the expression of SPARC transcripts. The relative amount of the PCR product (AU, arbitrary units) amplified from control liver samples was set at 1. *p<0.05 versus control (−TAA), **p<0.01 versus control (−BDL), Mann-Whitney test.

We first investigated whether SPARC expression levels may be upregulated in the liver of cirrhotic patients. To address this issue, liver biopsies taken from cirrhotic and non-cirrhotic patients were processed for qPCR studies. A significant upregulation in SPARC expression levels was observed in cirrhotic samples when compared to non-fibrotic ones (Figure 1A). These data suggest a possible role for SPARC in liver fibrogenesis.

**Figure 2:**
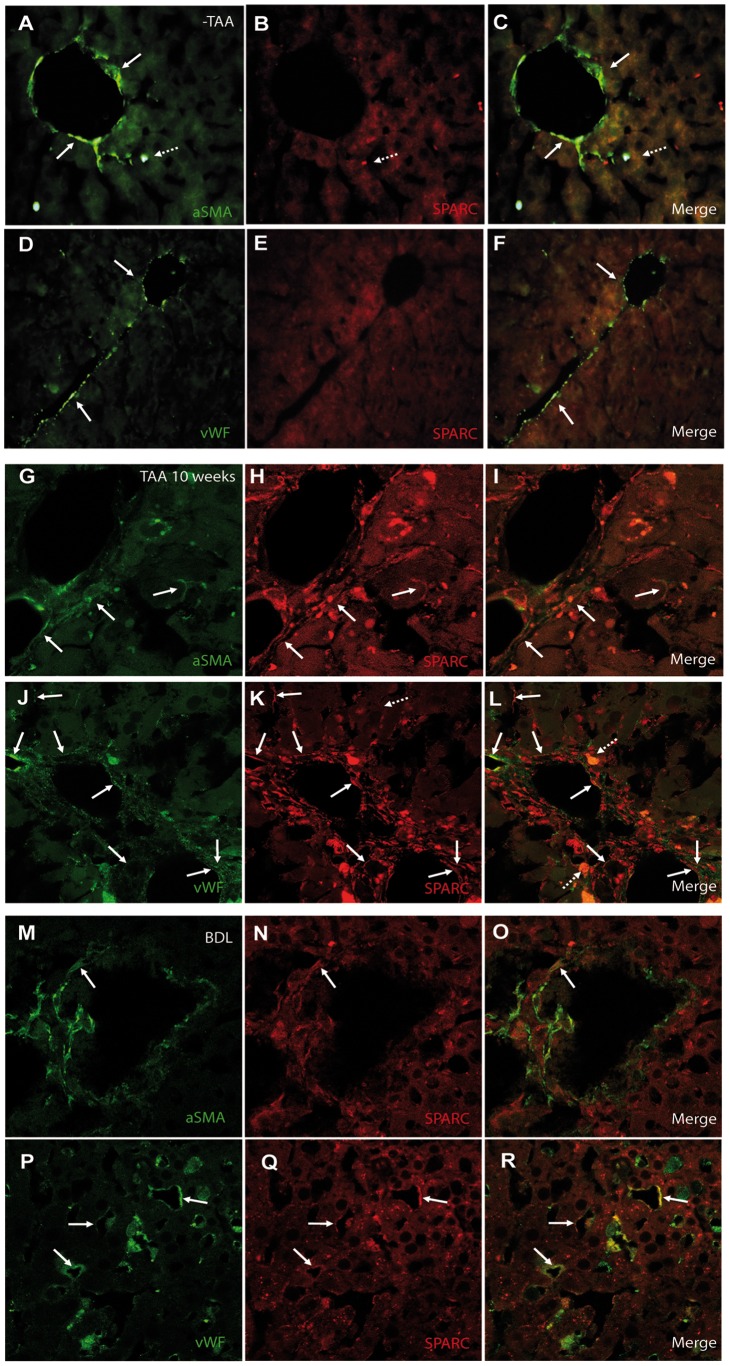
Patterns of SPARC expression during liver fibrogenesis. (A–F) Representative images taken from SPARC^+/+^ mice liver sections stained for SPARC (B, E; red) and SMA (A; green) or vWF (D; green) and merge of both images (C, F). (G–L). Representative images taken from 10 weeks TAA-treated SPARC^+/+^ mice liver sections (n  = 4–6) stained for SPARC (H, K; red) and SMA (G; green) or vWF (J; green). Co-localization of SPARC and SMA (I) or SPARC and vWF (L). (M–R) Representative images taken from 7 days BDL SPARC^+/+^ mice liver stained for SPARC (N, Q; red) and SMA (M; green) or vWF (P; green). Arrows: co-expression of the two markers; dotted arrows: autofluorescence due to hepatic ceroid-laden macrophages. Original magnification 400X (A–F) or 1000X (G–R).

We next asked whether or not SPARC expression could be similarly induced in different *in vivo* models developed in SPARC^+/+^ mice. To address whether SPARC expression levels may change during liver fibrogenesis, samples were processed for qPCR studies. SPARC was found to be induced after 2 weeks of TAA treatment and its expression levels remained similar after 10 weeks of treatment, as well as in mice subjected to BDL (Figure 1B). While in non-treated animals SPARC expression was undetectable (Figure 2 B and E), after 10 weeks of TAA treatment, SPARC was additionally expressed in fibrous septae as well as in parenchymal areas, surrounding sinusoids (Figure 2H and K). SPARC was found to be expressed by α-SMA^+^ myofibroblast cells, mainly within fibrous septae (Figure 2G–I). In addition, SPARC was also found to be expressed by vWF^+^ endothelial cells (Figure 2J–and L). A similar expression pattern was observed in the liver of mice subjected to BDL. SPARC was expressed by α-SMA^+^ myofibroblast cells mainly in the portal areas, but most of the expression was observed in endothelial cells (Figure 2M–R). From these results we can conclude that SPARC is overexpressed in the liver of cirrhotic patients and of mice exposed to different stimuli inducing fibrogenesis.

### Decreased Liver Fibrosis and Necroinflammation in SPARC Deficient Mice

**Figure 3:**
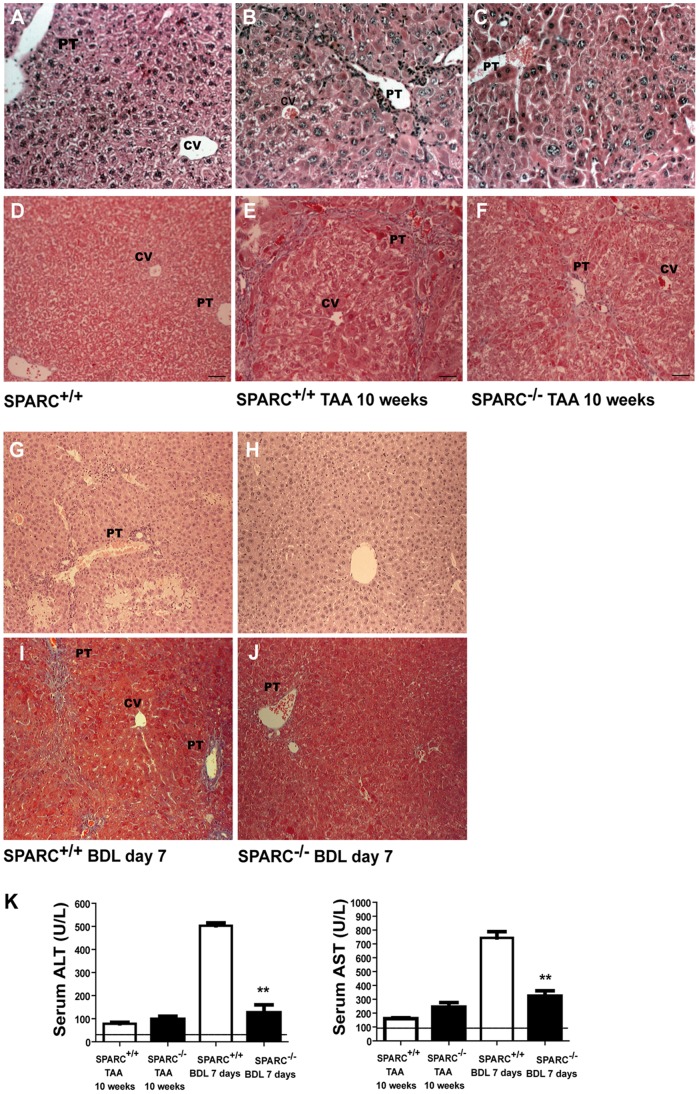
Reduced liver damage in SPARC deficient mice. (A–F) Representative photomicrographs of liver sections from untreated SPARC^+/+^ (A) or 10 weeks TAA-treated SPARC^+/+^ or SPARC^−/−^ mice (n  = 6–8), stained with H&E (A–C) or Masson’s trichrome (D–F). (G–J) Representative photomicrographs of liver sections from SPARC^+/+^ and SPARC^−/−^ mice subjected to BDL, stained with H&E (G–H) or Masson’s trichrome (I–J). Original magnification 200X. PT, portal tract; CV, central vein. (K) Serum ALT and AST levels were measured at the indicated time in TAA-treated and BDL mice. Dotted lines, upper normal limited. **p<0.05 versus treated SPARC^+/+^.

We next decided to investigate whether SPARC deficiency may influence liver fibrogenesis and processes therein involved. With this aim, two types of liver fibrosis *in vivo* models were applied to SPARC^−/−^ and in SPARC^+/+^ mice: chronic TAA application and bile duct ligation. After 10 weeks of TAA administration, SPARC^+/+^ livers showed extensive appearance of portal-portal and central-portal fibrous septae, regenerative nodules, and distortion of liver architecture (Figures 3). However, a marked reduction in the amount of fibrous septae and of regenerative nodules was found in TAA-treated SPARC^−/−^ animals (Figure 3A–F). Similar results were observed in animals subjected to BDL at 7 days post-surgery: while SPARC^+/+^ mice developed prominent fibrous expansions in periportal areas, they were almost absent in SPARC^−/−^ animals (Figure 3G–J).

Chronic liver injury and fibrogenesis are intimately linked to hepatocyte death and inflammation. To further study whether SPARC deficiency may induce changes in these mechanisms during liver fibrogenesis, these features were analyzed by an expert pathologist. A significant reduction in the periportal or periseptal interface hepatitis, focal necrosis and portal inflammation were observed in TAA-treated SPARC^−/−^ when compared to TAA-treated SPARC^+/+^ mice (Figure 3A–F and Table 1). All these differences contributed to the significant reduction in the Knodell score, frequently used to evaluate necroinflammatory activity, obtained from SPARC deficient fibrotic liver sections when compared to wild type fibrotic control tissue (Table 1). Similar results were obtained in BDL SPARC^−/−^ mice although the amount of portal inflammation was similar in comparison with SPARC^+/+^ mice.

**Table 1:** Severity of necroinflamatory activity and fibrosis in SPARC^+/+^ and SPARC^−/−^ mice after 10 weeks of TAA treatment (n  = 6–8) or in SPARC^+/+^ and SPARC^−/−^ BDL mice (n  = 5–6).

	SPARC^+/+^	SPARC^−/−^	SPARC^+/+^	SPARC^−/−^
	TAA 10 weeks	TAA 10 weeks	BDL 7 days	BDL 7 days
Periportal or Periseptal Interface hepatitis	2.7±0.5	1.3±0.4*	1.6±0.5	0.62±0.2
Focal (spotty) lytic necrosis, focal necrosis, apoptosis and focal inflammation	4±1.4	2.3±0.5*	3.5±0.3	0.75±0.25*
Portal inflammation	2.5±0.6	1.16±0.9*	2±0.0	1.5±0.3
Fibrosis	3.7±0.5	2.5±0.5*	2.6±0.2	0.5±0.0*
Knodell score	13.25±1.2	7.33±1.5*	9.75±0.5	3.25±0.3*

* p<0.05.

The present study has shown that there was a significant increase in aspartate transaminase (AST) and alanine transaminase (ALT) levels in SPARC^+/+^ BDL mice in comparison with BDL SPARC^−/−^. No significant differences were observed in SPARC^+/+^ and SPARC^−/−^ mice in TAA groups (Figure 3K) suggesting that the inflammatory stimuli generated by TAA intoxication is strong during the first weeks of administration and correlates with elevated levels of transaminases but not at week 10, when the fibrotic changes predominate over necrosis of hepatocytes.

**Figure 4:**
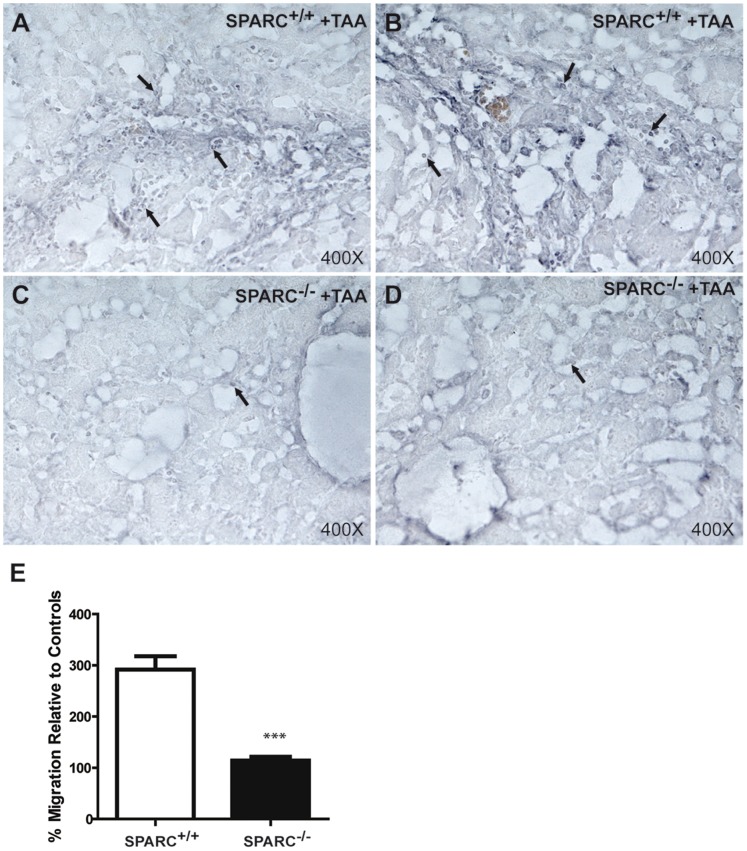
Reduced hepatic inflammatory infiltration and migratory capacity in SPARC deficient mice. Photomicrographs of representative liver sections from TAA-treated animals. SPARC^+/+^ mice showed an increased CD4^+^ cells infiltration in hepatic parenchyma, especially around portal tracts (A,B); while in TAA-treated SPARC^−/−^ mice CD4^+^ cells are scarce and located near the sinusoids (C,D). Arrows indicate CD4^+^ cells. Original magnification 400X. (E) Migratory response of splenocytes towards CCL19 chemokine. Percentage of cells that migrated relative to respective controls for SPARC^+/+^ and SPARC^−/−^ splenocytes (n = 3 replicates) in a Boyden Chamber system. Splenocytes were placed into the upper well, separated from the lower by a 5-µm porosity membrane. The bottom well contained either DMEM or DMEM with 10 ng/µl rCCL19 and cells were allowed to migrate during 2 h. ***p<0.001, Mann Whitney test.

In order to characterize the profile of immune cells in the hepatic inflammatory infiltrate we performed immunohistochemistry for CD4+ T cells and observed that the amount of CD4+ cells was greatly decreased in SPARC^−/−^ mice (Figure 4 A–D). Migration ability towards rCCL19 chemokine was explored in splenocytes derived from SPARC^−/−^ and SPARC^+/+^ mice in vitro. We observed a reduced migration in SPARC^−/−^ mice in response to CCL19 after 2 h of incubation (Figure 4E). Flow cytometry analysis of splenocytes showed similar expression of CCR7 receptor (not shown). In agreement with the microarrays results showing a decreased expression of CCL19 in SPARC^−/−^ mice (Table 2), qPCR assay confirmed down-regulation of the transcript (Figure S2).

**Table 2:** IPA® top molecules which were significantly alters in SPARC*^−/−^* versus SPARC^+/+^ mice and those modified in SPARC*^−/−^* versus SPARC^+/+^10 weeks TAA treated mice.

IPA® Top Molecules. Background		
Fold change up-regulated		
	Gene ID	Exponential Value
NIPAL1	NM_001081205	1,975
ACCN5	NM_021370	1,608
PIK3C2G	NM_207683	1,561
LOXL4	NM_001164311	1,514
SLC34A2	NM_011402	1,232
C17orf78	NM_001037932	1,230
UBE2U	NM_001033773	1,171
NDRG1	NM_008681	1,118
THEM5	NM_025416	1,117
FOXQ1	NM_008239	1,102
**Fold change down-regulated**		
	**Gene ID**	**Exponential Value**
SPARC	NM_009242	−4,051
C1orf51	BC132471	−2,245
USP2	NM_198092	−1,994
CCL19	NM_011888	−1,544
TSKU	NM_001168541	−1,508
CABYR	NM_027687	−1,338
PPIH	NM_001110130	−1,335
PER2	NM_011066	−1,120
P2RY2	NM_008773	−0,984
SYDE2	NM_001166064	−0,892
**IPA® Top Molecules. Ten Weeks of TAA Treatment**		
**Fold change up-regulated**		
	**Gene ID**	**Exponential Value**
NPY	NM_023456	2,092
CYP1A1	NM_009992	2,053
1600029D21Rik	NM_029639	2,028
Sprr1a	NM_009264	1,615
SLC7A11	NM_011990	1,498
CLDN4	NM_009903	1,487
ARG2 (includes EG:11847)	NM_009705	1,471
MCM6	NM_008567	1,449
TMC5	NM_001105252	1,358
TMEM45A	NM_019631	1,287
**Fold change down-regulated**		
	**Gene ID**	**Exponential Value**
SPARC	NM_009242	−4,517
UBLCP1	NM_024475	−2,273
CIDEC	NM_178373	−1,723
AKR1C3	NM_134066	−1,659
ACOT2	NM_134188	−1,637
CHRNA2	NM_144803	−1,223
SLC22A5	NM_011396	−1,097
CYP8B1	NM_010012	−1,022
CPT1B	NM_009948	−1,002
LYVE1	NM_053247	−0,965

### SPARC Deficient Mice Showed a Decreased Collagen Deposition

**Figure 5:**
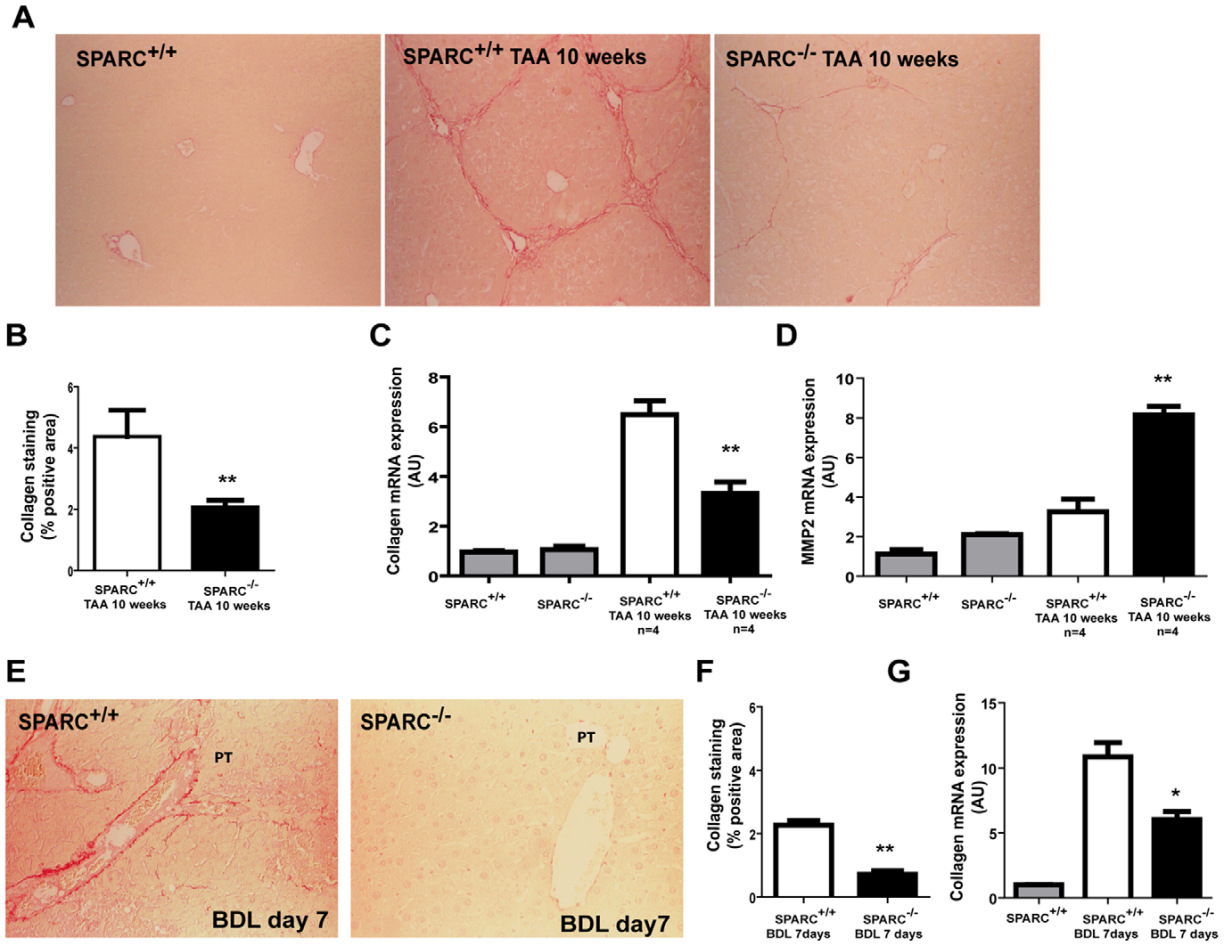
Reduced liver fibrosis in SPARC deficient mice. (A) Representative photomicrographs of liver sections stained with picrosirius red. SPARC^+/+^ mice show staining limited to periportal areas (left panel), while liver sections from TAA-treated SPARC^+/+^ mice exhibits marked portal fibrosis and portal-portal bridges (central panel) and those from TAA-treated SPARC^−/−^ mice present weak fibrotic response (right panel). (B) Morphometric quantification of Sirius red stained area showing a significant attenuation of the fibrotic process in TAA-treated SPARC^−/−^ mice when compared to treated wild-type mice. **p<0.01, Mann-Whitney test. (C–D) Quantitative data from qPCR analysis of collagen (COL1A2) and MMP-2 mRNA expression. *p<0.05, **p<0.01 versus SPARC^+/+^ TAA 10 weeks, Mann-Whitney test. (E) Representative pictures taken from liver sections of from SPARC^+/+^ or SPARC^−/−^ mice, at 7 days after BDL. Original magnification 200X. PT, portal tract; CV, central vein. (F) Morphometric quantification of Sirius red stained area showing a significant attenuation of the fibrotic process in SPARC^−/−^ mice at day 7 after BDL when compared to wild-type mice. **p<0.01, Mann-Whitney test. (G) qPCR analysis of collagen mRNA expression in SPARC^+/+^ and SPARC^−/−^ mice subjected to BDL. *p<0.05, versus BDL SPARC^+/+^ Mann-Whitney test.

In order to quantify liver content of collagen, the ECM protein most abundantly accumulated in fibrous septae, tissue sections from 10 weeks TAA-treated animals were Sirius red stained and morphometric analysis was thereafter performed. A significant reduction in Sirius red^+^ area was found in SPARC^−/−^ when compared to SPARC^+/+^ mice (Figure 5A–B). Consistently, α2(I) collagen mRNA expression levels were significantly reduced in SPARC deficient when compared to wild-type animals (3.31±0.64 vs. 6.48±0.95; SPARC^−/−^ vs. SPARC^+/+^) (Figure 5C). Matrix metalloproteinases (MMPs) are known to be involved in ECM regulation. We observed that hepatic MMP-2 expression was significantly increased in SPARC^−/−^ TAA-treated mice in comparison with SPARC^+^/^+^ (8.15±0.42 vs. 3.25±0.64, respectively) (Figure 5D).

In accordance with previous results, Sirius red staining on liver tissue and morphometric analysis obtained from BDL SPARC^−/−^ mice showed a remarkable decrease in the appearance of collagen deposits when compared with similarly treated control animals. While very little Sirius red staining was observed in SPARC deficient mice, abundant collagen deposits were found within fibrous expansions in periportal areas as well as in liver parenchyma of SPARC^+/+^ mice (Figure 5E–F). Consistent with Sirius red staining, α2(I) collagen mRNA expression levels were significantly reduced in SPARC deficient when compared to wild-type animals (6.03±0.67 vs. 10.86±1.08; SPARC^−/−^ vs. SPARC^+/+^).

### SPARC Deficiency Results in Immature Collagen Fibers Appearance and Packaging in Fibrotic Livers and in a Decrease Deposition of Hyaluronic Acid

**Figure 6:**
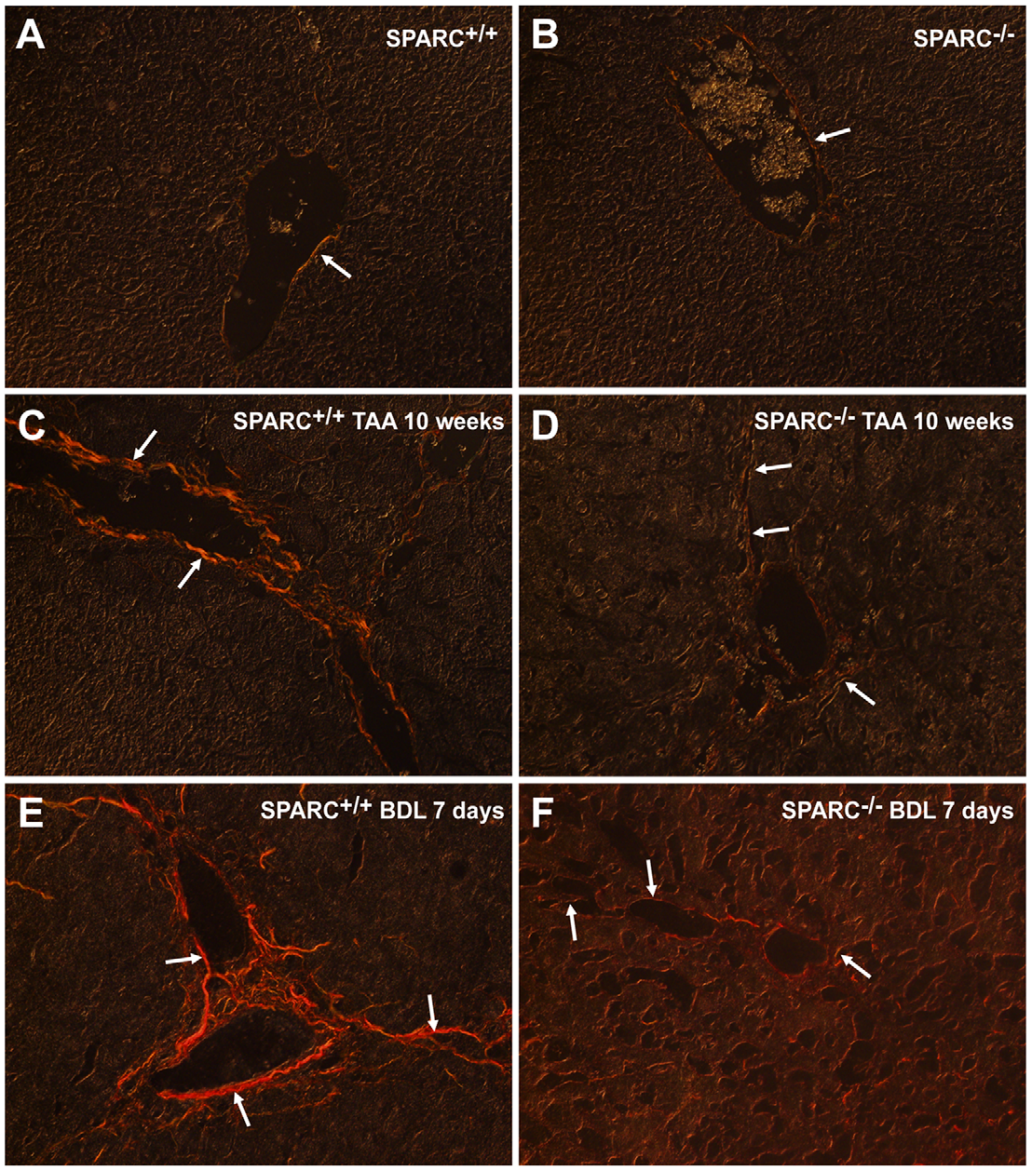
Reduced maturation of SPARC^−/−^ collagen fiber deposits. Representative pictures showing picrosirius red stained liver sections obtained from SPARC^+/+^ (A,C,E) or SPARC^−/−^ (B,D,F) mice (n  = 6–8) observed under polarized light. Animals were left untreated (A,B), or were TAA-treated during 10 weeks (C,D) or subjected to BDL (E, F). Note the predominant mature and compacted nature of collagen fibers in wild-type treated mice and their immature and thin appearance in SPARC^−/−^ animals. Original magnification 400X.

In SPARC^−/−^, both TAA and BDL resulted in Sirius red stained fibers which in most cases could not be observed under polarized light due to their reduce thickness and immature state: in this setting they turned into green which make very difficult to distinguish them from the overall tissue (Figure 6). On the other hand, most of Sirius red stained collagen fibers observed in TAA and BDL SPARC^+/+^ mice could be also observed under polarized light, due to their increase in thickness and in maturation state: in this case they became orange to red in color after applying this procedure.

Hyaluronic acid accumulation in the liver is considered a sign of advanced liver fibrogenesis [25]. Consistent with previous results, TAA-treated SPARC^+/+^ mice livers showed significant deposition of hyaluronan within fibrous septae while it was almost absent in TAA-treated SPARC^−/−^ animals (Figure S1).

### Reduced Number of Activated Myofibroblasts in Fibrotic Livers from SPARC^−/−^ Mice

**Figure 7:**
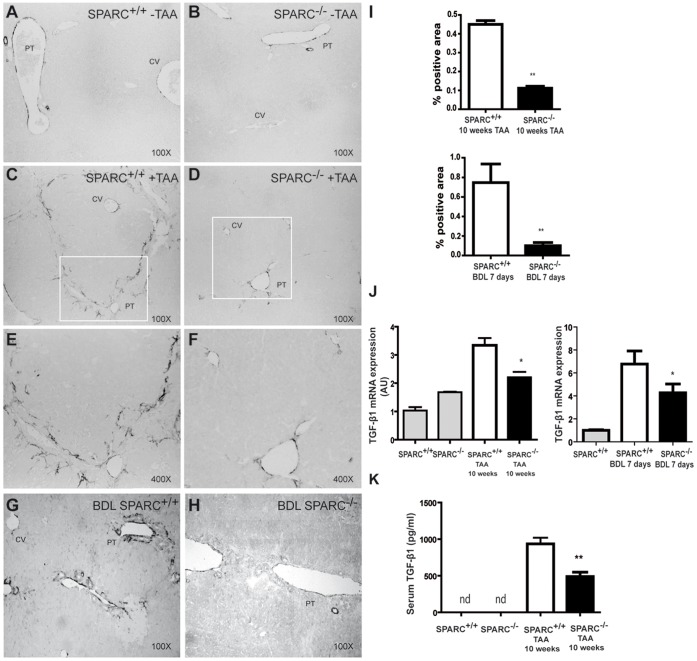
Reduction in the number of active myofibroblasts and in liver and serum TGF-β1 levels in SPARC deficient fibrotic mice. (A–H) Representative pictures taken from liver sections of untreated SPARC^+/+^ and SPARC^−/−^ (A,B), 10 weeks TAA treated SPARC^+/+^ (C,E) andSPARC^−/−^ (D,F), or BDL SPARC^+/+^ (G) and SPARC^−/−^ (H) mice immunostained for α-SMA. (E,F) are higher magnification images from box areas in C,D respectively. (I) Quantitative data of densitometric analyses of αSMA immunostained area from TAA-treated and BDL SPARC^−/−^ or SPARC^+/+^ mice. **p<0.01, Mann-Whitney test. (J) Quantitative data of TGF-β1 mRNA levels obtained by qPCR analysis from 10 weeks TAA-treated and BDL SPARC^+/+^ or SPARC^−/−^ mice (n  = 6–8). Data are expressed as relative values to those of wild-type mice without treatment. *p<0.05, SPARC^+/+^ treated vs SPARC^−/−^ treated. Mann-Whitney test. (K) Serum levels of TGF-β1 were measured after 10 weeks of TAA treatment. nd, non-detectable. **p<0.01, Mann-Whitney test.

Liver fibrogenesis is characterized by trans-differentiation of different cells into myofibroblasts, including HSCs. HSC-derived myofibroblasts are known to upregulate their α-SMA expression levels during the activation process. In order to address whether either the number of myofibroblasts in fibrous septae and/or the activation state of myofibroblasts might be affected by SPARC deficiency, liver tissue obtained from TAA-treated and BDL mice was immunostained with α-SMA. A significant reduction in the α-SMA^+^ immunostained area was found in TAA-treated SPARC^−/−^ when compared to TAA-treated SPARC^+/+^ mice (0.11±0.01 vs. 0.45±0.02, respectively) (Figure 7A–F and 7I) and in SPARC^−/−^ subjected to BDL compared to SPARC^+/+^ mice (0.1±0.03 vs. 0.7±0.19, respectively) (Figure 7G–H and 7I). These results suggest that a reduction in the number of activated myofibroblasts is likely involved in the inhibition of liver fibrogenesis found in SPARC deficient mice.

### Downregulation of TGF-β1 Hepatic Expression and Systemic Levels in TAA Treated SPARC Deficient Mice

SPARC is known to be involved in a positive autocrine feedback loop with TGF-β1, a fibrogenic cytokine with a crucial role in liver fibrosis. In order to analyze whether SPARC deficiency may cause downregulation of TGF-β1 expression, its mRNA expression levels in fibrotic liver from 10 weeks TAA-treated and BDL mice were measured by qPCR. A significant decrease in the expression of this cytokine was found in SPARC deficient mice when compared to control (Figure 7J). To further confirm these results, serum levels of TGF-β1 were measured by ELISA. Significant lower levels of TGF-β1 were found in samples obtained from 10 weeks TAA-treated SPARC^−/−^ when compared to those from TAA-treated SPARC^+/+^ mice (Figure 7K). These results suggest that anti-fibrotic effects of SPARC deficiency are likely partially mediated by downregulation of TGF-β1 expression levels.

### Microarray Analyses Show Changes in Gene Expression Profile in Naïve SPARC Deficient Mice

In order to explore whether lack of SPARC expression might influence liver gene expression profile in late fibrogenic processes, cDNA expression arrays were performed and analyzed (Affymetrix Mouse Gene ST 1.0 array). To analyze a possible involvement of basal physiological conditions (“background” effects) in the liver of SPARC deficient or wild-type mice which might partially explain the differences observed in liver fibrosis models, we have first compared gene expression profiles among samples of untreated animals. The genes responsible for basal effect should be found up- or down- regulated.

**Figure 8:**
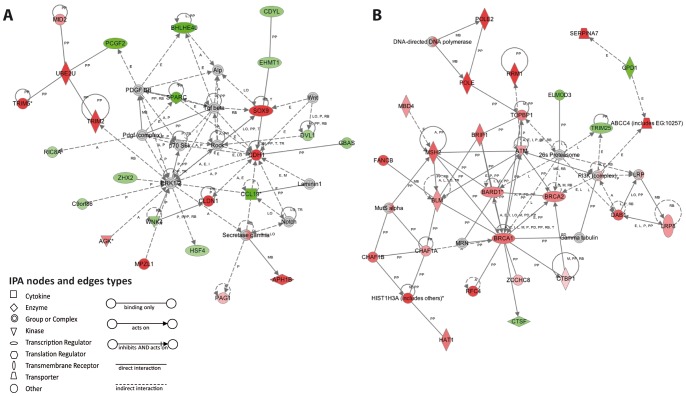
The top network of differentially expressed genes. The networks are presented as graphical displays where genes appear as nodes and the molecular relationships are represented by lines. Up-regulated and down-regulated genes in SPARC*^−/−^* mice are shown as red spot or green spot, respectively. The top network of differentially expressed genes in SPARC*^−/−^* versus SPARC^+/+^ mice (A) or SPARC*^−/−^* after 10 weeks of TAA treatment versus SPARC^+/+^ TAA treated mice (B), as identified by IPA analysis. Intensity of the red or green color shows the level of gene expression. Gray represents a gene found which is related to the others but did not meet the cutoff criteria. A Activation, E Expression (includes metabolism/synthesis for chemicals), I Inhibition, L Proteolysis (includes degradation for Chemicals), LO Localization, M Biochemical Modification, MB Group/complex Membership, P Phosphorylation/Dephosphorylation, PD Protein-DNA binding, PP Protein-Protein binding, RB Regulation of Binding,T Transcription,TR Translocation.

A total of 139 upregulated genes (124 known genes and 15 un-known cDNAs or ESTs) and 155 downregulated genes (138 known genes and 17 unknown cDNAs or ESTs) were obtained (Table S1). To analyze microarray data, three strategies were followed. Lists of the 10 top upregulated or downregulated gene lists (Table 2) and gene-interactions networks were performed (Ingenuity) (Figure 8), and modified genes were classified in ontological categories (GO, gene ontology) (Table S2). The analyses of the top up- or down- regulated showed relevant candidate genes in SPARC*^−/−^* mice compared to SPARC^+/+^ mice. It is worth to noting that LOXL4, an important protein involved in the regulation of extracellular matrix components [26], was found increased while USP2, previously involved in triggering hepatocyte apoptosis and CCL19, related with inflammation and fibrogenesis [27], [28], were down-regulated in SPARC*^−/−^* untreated liver tissues when compared to SPARC^+/+^ mice, likely suggesting an initial condition of liver cells which would make them less susceptible to death and compatible with a subsequent reduction in fibrosis development.

Ingenuity Pathway Analysis (IPA) analyses (p<0.01) suggested several gene networks which likely involve protective mechanisms of SPARC deficiency against liver fibrogenesis. The network with the highest score and biological significance was chosen (Figure 8A). In this model, lack of SPARC expression was found related to Sox9 expression. Thus, Sox9 (sex determining region Y-box 9) and CDH1 (Cadherin-1 or E-cadherin; with a gene expression activity regulated by and known to be able to bind to Sox9) were found up-regulated in the liver of SPARC*^−/−^* mice. In addition, other genes showing increased expression levels in these mice within the same gene interaction network were cldn1 (claudin 1), secretase gamma, aph1b (anterior pharynx defective 1b homolog), pag1 (phosphoprotein associated with glycosphingolipid microdomains 1), mpzl1 (myelin protein zero-like 1) and agk (acylglycerol kinase). In addition, lack of SPARC expression was found related to a down-regulation in PCGF2 (polycomb group ring finger), a factor that maintains repression of genes related to tumorigenesis and cell cycle [29]. In turn, PCGF2 likely interacts with UBE2U (ubiquitin-conjugating enzyme E2U), involved in ubiquitination, found upregulated in SPARC*^−/−^* mice. Consistently, other proteins able to interact with UBE2U, such as two members of the TRIM (tripartite motif-containing) family and MID2 (midline 2), were also found upregulated upon SPARC deficiency.

Interestingly, the GO analysis revealed statistically enriched ontological categories including those related in chromatin remodeling, cAMP signaling or cell cycle regulation. A complete list of all genes included in each GO category is given as supplementary information. Overall our data suggest that the liver of SPARC deficient animals have a gene expression profile which likely makes them less susceptible to develop liver fibrosis.

### Microarray Analyses Show Changes in Gene Expression Pattern in Advanced Liver Fibrogenesis due to SPARC Deficiency

To uncover new protective mechanisms involved in the observed reduction in the degree of liver fibrosis observed in SPARC deficient mice, cDNA expression array analyses were performed comparing liver tissue samples obtained from SPARC*^−/−^* and SPARC^+/+^ mice after 10 weeks of TAA administration. A total of 492 genes showed a p<0.01 (281 upregulated and 211 downregulated genes) in SPARC*^−/−^* mice. Top up-regulated and down-regulated genes are shown in Table 2. They include upregulation of CLDN4 (Claudin 4), a component of the tight junction strands, and downregulation of CIDEC (cell death-inducing DNA fragmentation factor-α-like effector C), a potent apoptosis inducer [30]. Interestingly, canonical pathway and biological functions identified by IPA showed a large group of upregulated genes associated with DNA repair and detoxification. Remarkably a large group of interacting genes were found upregulated (Figure 8B) in the context of SPARC deficiency including at the center of the network those encoding ATM (ataxia telangiectasia-mutated) kinase [31] and the transcription factors BRCA (breast cancer) 1 [32], BRCA2 [33] and BARD1 (BRCA1-associated RING domain) [34] and including effector genes such as topbp1 (topoisomerase (DNA) II binding protein 1) [31], rrm1 (ribonucleotide reductase M1) [35], brip1 (BRCA1 interacting protein C-terminal helicase 1) [36], pole1 (polymerase (DNA directed), epsilon, catalytic subunit) and 2 (polymerase (DNA directed), epsilon 2, accessory subunit) [37], msh2 (mutS homolog 2, colon cancer, nonpolyposis type 1 (E. coli) [38], mbd4 (methyl-CpG binding domain protein 4) [39], blm (Bloom syndrome, RecQ helicase-like) [38], fancb (Fanconi anemia, complementation group B) [40], rfc4 (replication factor C (activator 1) 4) [37], chaf1A (chromatin assembly factor 1, subunit A (p150) and B (chromatin assembly factor 1, subunit B (p60)) [41], Hist1H3A (histone cluster 1, H3a) and hat1 (histone acetyltransferase 1) [42]. Consistently, E2F, able to induce gene expression of many transcriptional factors and effectors in DNA repair and stability pathways including some of the most relevant ones mentioned above [43], was found upregulated at the center of the second most relevant IPA gene network map, as well as other related functional proteins such as PCNA (proliferating cell nuclear antigen), MCM2 (minichromosome maintenance deficient 2 mitotin), MCM3, MCM4 (minichromosome maintenance deficient 4 homolog), MCM6, MCM7, RPA2 (replication protein A2), PRIM1 (DNA primase, p49 subunit), PRIM2 (A primase, p58 subunit), CDT1 (chromatin licensing and DNA replication factor 1) and ASF1B (ASF1 anti-silencing function 1 homolog B); not shown). It is worth noting that the first and most significant gene network model showed an upregulation of ABCC4 (ATP-binding cassette sub-family C member 4), a key ABC transporter involved in the removal of chemicals, xenobiotics and products of oxidative stress [44], and the downregulation of TRIM25 (tripartite motif-containing 25) [45] and CTSF (cathepsin F) [46] involved in protein degradation processes.

## Discussion

SPARC expression is known to be induced during liver fibrogenesis in different species. In mouse, SPARC upregulation has only been reported in a schistosomiasis model of liver fibrosis [6]. In this work, we confirmed this result in two additional models of this disease caused by different etiologies: hepatotoxicity mediated by chronic TAA intoxication, and cholestasis induced by BDL. In addition, we provide new evidence, based on a genetic *in vivo* model, showing that SPARC, expressed by HSC and endothelial cells, is involved in liver fibrogenesis. Moreover, our data support that SPARC plays a major role in key pathogenic events related to the fibrogenic process such as hepatocyte necrosis, inflammation and recruitment/activation of myofibroblasts as well as in the induction of the profibrogenic cytokine TGF-β1. These findings are consistent with previous reports, based on studies performed with other tissues, showing SPARC involvement in proinflammatory and profibrogenic mechanisms [47], [48]. Our gene expression profile analyses suggest that SPARC upregulation in the fibrotic liver might induce deficiencies in DNA repair mechanisms likely resulting in enhanced liver cell death and subsequent increased ECM deposition as a consequence of scar formation process. Overall, our data strongly support that SPARC plays a prominent profibrogenic role in the context of chronic liver injures, providing new clues to understand mechanisms therein involved. Although gene targeting is a powerful tool for the study of a disease on a uniform genetic background, the gene knockout approach must be used with caution, particularly in interpretations of the phenotypes that are obtained in liver biology, where many genes have pleiotropic functions.

Myofibroblasts do normally accumulate in the parenchyma close to liver injured areas. Many of them most likely derive from resident HSCs, the main source of fibrillar collagen and the major targets of anti-fibrotic therapies [49]. By mean of immunohistochemical studies, a decrease in the number of α-SMA^+^ myofibroblasts was observed in fibrotic livers of SPARC deficient mice. This finding is consistent with our recently published data showing that specific SPARC knock-down in activated HSCs reduces their migratory ability as well as their activation state [50]. Thus, the induction of SPARC expression in activated HSCs likely promotes their accumulation in chronically injured liver areas further aggravating the pathologic state. Whether SPARC may or not participate in the activation/proliferation of other non-parenchymal cells such as Kupffer, endothelial or biliary epithelial cells in response to chronic injury is unknown and merits further investigation.

In addition, recent studies demonstrated that SPARC is able to exert potent stimulatory effects on TGF-β1 expression in cultured HSCs [5], skin and lung fibroblasts, and mesangial cells [4], [15], [51]. In agreement with this, it is worth noting that in these studies lower levels of TGF-β1 were found in fibrotic livers and in serum samples from TAA-treated SPARC deficient mice when compared to wild-type counterparts. Therefore, and in the context of fibrotic diseases, these data further emphasizes the prevailing hypothesis of TGF-β1 expression being dependent on local SPARC expression levels. Based on the abundant literature related to the function of TGF-β1 in fibrogenesis [52] we hypothesize that this mechanism likely explain most of the observed phenotype.

Collagen fibrillar formation is a critical step in fibrogenesis and SPARC is a well-known collagen-binding matricellular protein with a reported role in type I collagen packaging. Thus by mean of *in vitro* studies, Rentz *et al.* have shown that collagen I produced by SPARC*^−/−^* cells was not efficiently incorporated into detergent-insoluble fractions [53]. In agreement with this, we herein show that fibrotic livers obtained from SPARC deficient mice contain thin, dispersed and predominantly immature collagen fibers while they were thick, highly compacted and mature in those from SPARC^+/+^ animals. Therefore, our data are consistent with previous reports on SPARC function in the context of collagen fiber maturation and compaction [54]. Little information is available on the role of SPARC in human liver fibrosis: we (herein) and others [7] showed that SPARC is overexpressed in the liver of cirrhotic patients. Nevertheless, these new data obtained from a mouse genetic model, using different *in vivo* disease models, are consistent with a profibrogenic role of SPARC in the context of chronic liver disease. Moreover, SPARC is shown to likely mediate key events in liver fibrogenesis such as liver inflammation, induction of TGF-β1 expression levels and the accumulation of active myofibroblasts.

Matrix metalloproteinase-2 (MMP-2), a type IV collagenase, is upregulated in chronic liver disease and is considered a profibrotic factor [55]. However, recent evidence in *in vivo* animal models revealed that MMP-2 deficiency is associated with increased hepatic collagen type I expression and fibrogenesis [56]. In agreement with these results, we observed that MMP-2 messenger RNA was increased at week 10 of TAA treatment in SPARC^−/−^ mice. In addition, our microarray analysis also demonstrated that MMP-2 is significantly increased in SPARC^−/−^ TAA-treated mice (not shown), suggesting that this effect may be involved, at least in part, in the protective effect of SPARC knock-down on liver fibrogenesis.

In an effort to identify changes in gene expression profile, which could explain the observed protective mechanisms against TAA damage in SPARC^−/−^ mice in an established model of liver fibrosis, microarray analyses were performed. A number of genes were narrowed down; nevertheless, a number of candidates showed a significant difference in expression when comparing SPARC knockout and wild-type mice. Our overall results suggest that the reduction in liver fibrogenesis observed in SPARC deficient mice seems to be the result of a sum of several mechanisms rather than the effect of changes in a small group of specific genes. Accordingly to this, we chose the approach of comparing ingenuity networks rather than analyzing changes in individual gene expression.

Interestingly, the list of top modified genes in untreated mice showed differential regulation of certain gene categories which might partially explain a protective status against any insult as a result of SPARC deficiency: the specific involvement of several of these genes in liver pathologies will be analyzed in our future research. A candidate gene would be LOXL4, a target of TGF-β1 [26] found to cause reduction in TGFβ1-mediated cell motility of hepatoma cells. In addition, USP2 was found down-regulated in SPARC^−/−^ mice likely rendering hepatocytes less susceptible to TNF-mediated apoptosis [57]. Another top down-regulated gene was CCL19, a chemokine known to attract dendritic cells and lymphocytes [58], [59], [60] which might be involved in the reduction of inflammation observed in SPARC deficient mice after TAA treatment. However, no significant differences were observed in CCL19 gene expression at week 10 of TAA administration leading us to speculate that low expression levels of CCL19 in null mice at the beginning of the injury with TAA could be critical for the subsequent generation of inflammation, necrosis of hepatocytes and fibrogenesis. The magnitude of inflammatory infiltrates was markedly reduced in SPARC^−/−^ mice, especially CD4^+^ cells, and the decreased migratory capacity of their splenocytes towards CCL19 chemokine *in vitro* leading us to speculate that the decreased fibrosis observed in these animals could be mediated, at least in part, by lessening of an inflammatory effect. This observation was not confirmed in BDL model. These results are in agreement with Rempel *et al.* who consider that that SPARC^−/−^ mice have an impaired immune system [61].

Furthermore, CCL19 receptor is expressed in HSCs and its activation was previously shown to induce its migration capacity [62] a relevant feature involved in liver fibrogenesis [63]. Upon TAA long-term treatment, CIDEC, a known potent inducer of apoptosis [30], [64], is among the top down-regulated genes in SPARC deficient mice, likely cross-linking altered metabolism with programmed cell death in the TAA-induced fibrosis model.

Using the IPA tool we were able to find highly significant networks and pathways likely involved in the described functional phenotype. In fact, naïve SPARC^−/−^ and SPARC^+/+^ mice seem to significantly differ in their gene expression profiles. As previously discussed, a positive feedback between SPARC and TGF-β1 was experimentally established; in addition, it is speculated that SPARC might regulate the signaling pathway induced by TGF-β1 [65]. Our best IPA gene network model showed that SPARC depletion was associated with an increased in SOX9 expression levels, likely mediated by TGF-β1 signaling pathway. SOX9 is a transcription factor involved in liver development, shown to contribute to hepatic physiology preservation [66]. However, a previous work suggested that SOX9 has a role as a transcriptional regulator in fibrogenesis promoting extracellular matrix deposition [67].

Additionally, the shown network establishes a relationship with CDYL and EHMT1 genes, codifying for histone methylases and acetylases likely involved in SOX9 gene regulatory function. Furthermore, in our network model SOX9 is shown to interact and to induce E-cadherin gene expression, also found upregulated in the liver of naïve SPARC deficient mice. E-cadherin is an important epithelial cell-cell adhesion protein considered as a marker of non-activated HSCs [68]. Claudin 1, a tight junction protein, is another adhesion protein also found overexpressed in SPARC null mice and known to interact and mutually activate each other with E-cadherin. Tight junctions are confined to epithelial cells, forming continuous belts around cells and serving as a physical barrier to regulate transport through the paracellular space. Another important event shown in the gene network model is the decrease of CCL19 in SPARC*^−/−^* mice, likely involved in the observed reduction in lymphocyte recruitment and process of liver inflammation which normally characterizes liver fibrosis as previously discussed. In turn, the decrease in CCL19 gene activity is related with several transcriptional repressor and stress response factors (such as HSF4, ZHX2), linked to ERK1/2 pathway, also found down-regulated. SPARC*^−/−^* mice have an increased in secretase gamma (protease complex) and its protein complex partner APH1B, as well as PAG1 (a protein shown to be phosphorylated by secretase gamma), likely involving Notch signaling pathway-related changes. Secretase gamma and PAG1 might be also involved in the regulation of T cell activation. Overall our results support the concept of basal conditions in the context of SPARC deficiency making the liver less susceptible to external insults such as those causing liver fibrosis.

In addition to the influence of a basal phenotype condition, the results of pathway analyses on samples obtained from TAA treated animals suggest that SPARC deficient mice have a more intact or better established machinery to repair DNA or detoxify the body from external chemical stimuli as suggested by the remarkable upregulation in genes such as ATM, BRCA1, BRCA2 and BARD1. ATM is a sensor of DNA damage and phosphorylates p53 (tumor suppressor, which can activate apoptosis and arrest the cell cycle if DNA damage is not resolved) [69]. Additionally, ATM knockout affects cell survival and liver regeneration [70]. Consistently, BRCA1 complexed with BARD1 has tumor suppression properties and is involved on protein ubiquitination for degradation. Moreover, it needs to form a complex with BRCA2, also herein upregulated, to repair damaged DNA. It is reported that TGFβ1/Smad3 counteracts BRCA1-mediated DNA repair [71] and expression [72]. Our results on upregulation in BRCA1 as well as in its related partners and effectors involved in DNA repair are consistent with the decreased in TGF-β1 levels found in our study. Via PI3K, ATM likely up-regulates ABCC4, a key ABC transporter involved in the removal of chemicals, xenobiotics and products of oxidative stress, as well as the efflux of GSH and bile salts through the basolateral membrane of hepatocytes [73]. Exported GSH may be mediating protection against oxidative damage in the tissue. It was previously reported that the expression and localization of this transporter in centrilobular hepatocytes was increase in mice receiving hepatotoxic doses of acetaminophen or CCl_4_ [74], which correlates with protection against further doses of acetaminophen [75]. Therefore, upregulation in the expression of ATM and of its related BCRA transcriptional factor complex components as well as of their specific effectors suggest the establishment of a more efficient machinery mounted to repair DNA defects likely resulting in less susceptibility to liver cell apoptosis. Such mechanisms together to detoxifying activities mediated by ABCC4 might explain the reduction in liver damage and subsequent fibrosis observed in SPARC deficient mice.

In summary, we herein show that SPARC depletion in a mouse genetic model results in protection against liver fibrosis development. Mechanisms therein involved are complex and likely act at different levels and through diverse processes implicated in fibrogenesis. Thus, our new evidences implicate reduction in the extent of liver inflammation, in TGF-β1 expression levels (a master molecule involved in the ECM deposition) and in the activation status of HSCs, and an increase in MMP2 expression. In addition, they suggest the establishment of a transcriptional activation of the DNA repair machinery making liver cells less susceptible to apoptosis thus likely preventing liver damage. Overall, giving the complexity of advanced liver fibrosis it is believed that therapeutic avenues need also to act at different parallel mechanisms to be efficient in ameliorating disease mechanisms. These studies further support a beneficial effect of SPARC downregulation and suggest that this might be a good candidate for an *in vivo* approach using gene therapy tools (e.g. long-term expression vectors) for future antifibrotic therapeutic actions.

## Supporting Information

Figure S1 **SPARC deficiency shows decrease deposition of hyaluronic acid.** Representative pictures taken from liver sections of TAA untreated (A, B) or 10 weeks TAA treated SPARC^+/+^ (C) or SPARC^−/−^ (D) mice stained for hyaluronic acid (n  = 6–8). Original magnification 100X. PT, portal tract; CV, central vein.
(TIF)

Figure S2 **qPCR analysis for selected microarray genes.** mRNA expression levels in liver samples of SPARC^+/+^ and SPARC^−/−^ mice, untreated or treated 10 weeks with TAA, of: (A) CCL19, (B) Brca1, (C) ABCC4F, (D) Cldn1, (E) ATMF, (F) Cdh1, (G) Bard1. Values were normalized to levels of GAPDH transcript. Error bars represent SD values. p values are presented in the figure.
(TIF)

Table S1 **List of the significantly upregulated and downregulated genes between SPARC^−/−^ and SPARC^+/+^, 6 weeks TAA treated SPARC^+/+^ and TAA treated SPARC^−/−^, 10 weeks TAA treated SPARC^+/+^ and TAA treated SPARC^−/−^.**
(XLSX)

Table S2 **List of the significantly modified gene classified by ontological categories.**
(XLS)
