# Supplementary figures and images for: Lack of the Matricellular Protein SPARC (Secreted Protein, Acidic and Rich in Cysteine) Attenuates Liver Fibrogenesis in Mice
Source: PLoS One. 2013 Feb 11;8(2):e54962. doi: 10.1371/journal.pone.0054962 (PMC3569438; doi:10.1371/journal.pone.0054962)

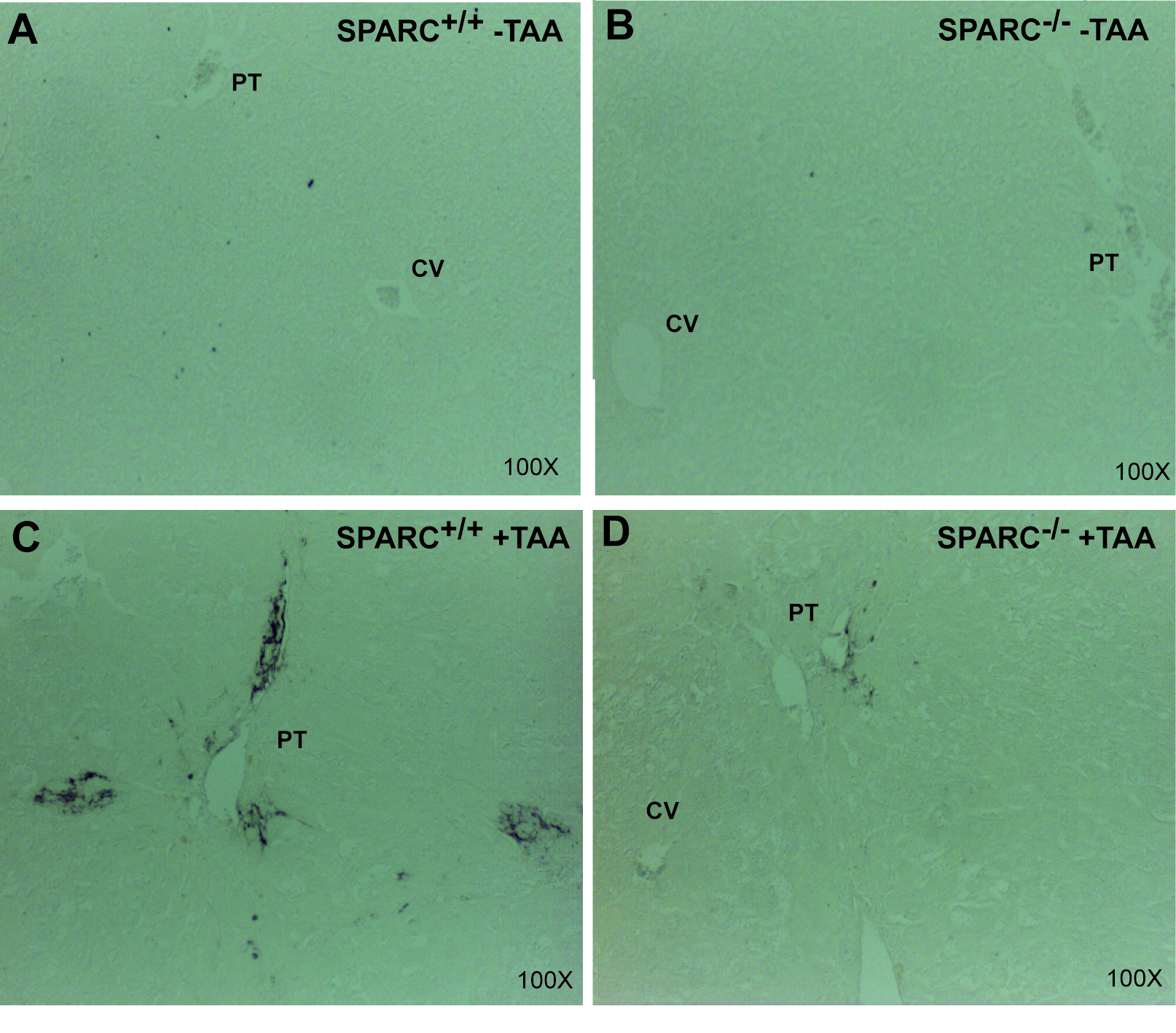

Supplement: Figure S1 — SPARC deficiency shows decrease deposition of hyaluronic acid. Representative pictures taken from liver sections of TAA untreated (A, B) or 10 weeks TAA treated SPARC+/+ (C) or SPARC−/− (D) mice stained for hyaluronic acid (n = 6–8). Original magnification 100X. PT, portal tract; CV, central vein. (TIF) [file pone.0054962.s001.tif]

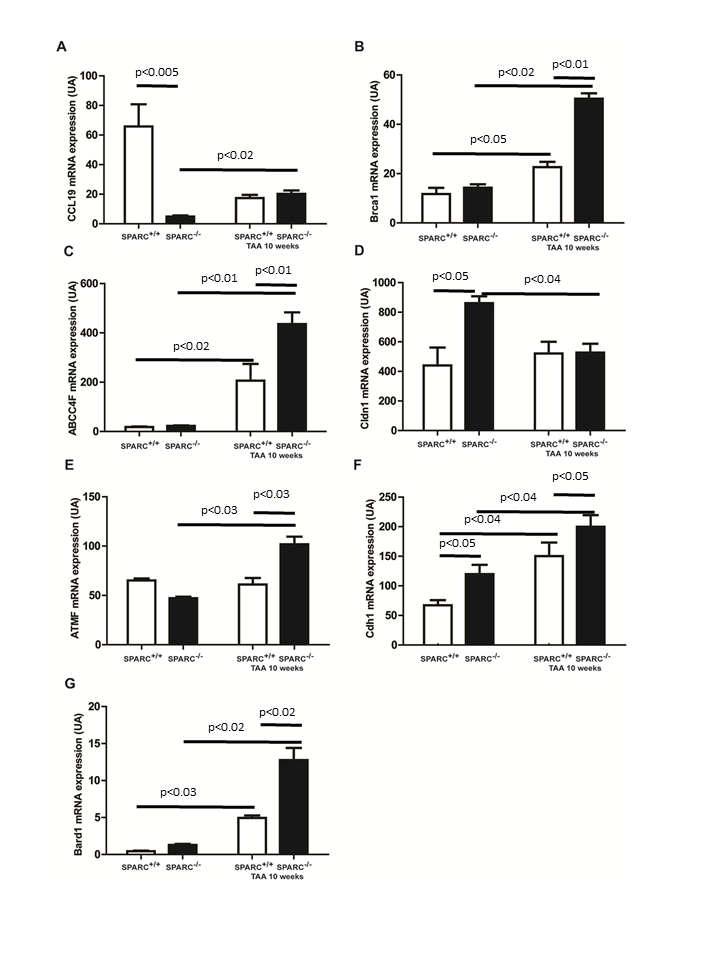

Supplement: Figure S2 — qPCR analysis for selected microarray genes. mRNA expression levels in liver samples of SPARC+/+ and SPARC−/− mice, untreated or treated 10 weeks with TAA, of: (A) CCL19, (B) Brca1, (C) ABCC4F, (D) Cldn1, (E) ATMF, (F) Cdh1, (G) Bard1. Values were normalized to levels of GAPDH transcript. Error bars represent SD values. p values are presented in the figure. (TIF) [file pone.0054962.s002.tif]
